# Antibiotic-Resistant *Arcobacter* spp. in commercial and smallholder farm animals in Asante Akim North Municipality, Ghana and Korogwe Town Council, Tanzania: a cross-sectional study

**DOI:** 10.1186/s13099-023-00588-3

**Published:** 2023-12-02

**Authors:** Ellis Kobina Paintsil, Linda Aurelia Ofori, Charity Wiafe Akenten, Andreas E. Zautner, Joyce Mbwana, Neyaz Ahmed Khan, John P. A. Lusingu, Joseph Kaseka, Daniel T. R. Minja, Samwel Gesase, Anna Jaeger, Maike Lamshöft, Jürgen May, Kwasi Obiri-Danso, Ralf Krumkamp, Denise Dekker

**Affiliations:** 1 Kumasi Centre for Collaborative Research in Tropical Medicine (KCCR), South-End, Asuogya Road, 039-5028 Kumasi, Ghana; 2 Department of Theoretical and Applied Biology, Kwame Nkrumah University of Science and Technology, 039-5028 Kumasi, Ghana; 3 Department of Implementation Research, One Health Bacteriology Group, Bernhard Nocht Institute for Tropical Medicine (BNITM), Bernhard-Nocht-Str. 74, 20359 Hamburg, Germany; 4 Institute of Medical Microbiology and Hospital Hygiene, Medical Faculty, Otto-Von-Guericke University Magdeburg, 39120 Magdeburg, Germany; 5 National Institute for Medical Research (NIMR), Tanga Centre, Tanga, Tanzania; 6 Bernhard Nocht Institute for Tropical Medicine (BNITM), Bernhard-Nocht-Str. 74, 20359 Hamburg, Germany; 7 German Centre for Infection Research (DZIF), Partner Site Hamburg-Lübeck-Borstel-Riems, 20359 Hamburg, Germany; 8 Tropical Medicine II, University Medical Center Hamburg-Eppendorf (UKE), 20251 Hamburg, Germany

**Keywords:** *Arcobacter*, Commercial farms, Smallholder farms, Antimicrobial resistance, Ghana, Tanzania, *Arcobacter butzleri*, *Arcobacter lanthieri*, *Arcobacter cryaerophilus*

## Abstract

**Background:**

*Arcobacter* species are considered emerging foodborne pathogens that can potentially cause serious infections in animals and humans. This cross-sectional study determined the frequency of potentially pathogenic *Arcobacter* spp. in both commercial and smallholder farm animals in Ghana and Tanzania. A total of 1585 and 1047 (poultry and livestock) samples were collected in Ghana and Tanzania, respectively. Selective enrichment media, along with oxidase and Gram testing, were employed for isolation of suspected *Arcobacter* spp. and confirmation was done using MALDI-TOF MS. Antibiotic susceptibility was assessed through disk diffusion method and ECOFFs were generated, for interpretation, based on resulting inhibition zone diameters.

**Results:**

The overall *Arcobacter* frequency was higher in Ghana (7.0%, n = 111) than in Tanzania (2.0%, n = 21). The frequency of *Arcobacter* in commercial farms in Ghana was 10.3% (n/N = 83/805), while in Tanzania, it was 2.8% (n/N = 12/430). *Arcobacter* was detected in only 3.6% (n/N = 28/780) of the samples from smallholder farms in Ghana and 1.5% (n/N = 9/617) of the samples from Tanzania. For commercial farms, in Ghana, the presence of *Arcobacter* was more abundant in pigs (45.1%, n/N = 37/82), followed by ducks (38.5%, n/N = 10/26) and quails (35.7%, n/N = 10/28). According to MALDI-TOF-based species identification, *Arcobacter butzleri* (91.6%, n/N = 121/132), *Arcobacter lanthieri* (6.1%, n/N = 8/132), and *Arcobacter cryaerophilus* (2.3%, n/N = 3/132) were the only three *Arcobacter* species detected at both study sites. Almost all of the *Arcobacter* from Ghana (98.2%, n/N = 109/111) were isolated during the rainy season. The inhibition zone diameters recorded for penicillin, ampicillin, and chloramphenicol allowed no determination of an epidemiological cut-off value. However, the results indicated a general resistance to these three antimicrobials. Multidrug resistance was noted in 57.1% (n/N = 12/21) of the *Arcobacter* isolates from Tanzania and 45.0% (n/N = 50/111) of those from Ghana. The type of farm (commercial or smallholder) and source of the sample (poultry or livestock) were found to be associated with multi-drug resistance.

**Conclusions:**

The high levels of MDR *Arcobacter* detected from farms in both countries call for urgent attention and comprehensive strategies to mitigate the spread of antimicrobial resistance in these pathogens.

**Supplementary Information:**

The online version contains supplementary material available at 10.1186/s13099-023-00588-3.

## Background

*Arcobacter* species are considered emerging foodborne pathogens that can potentially cause human infections [1, 2]. *Arcobacter* is closely related to *Campylobacter* in terms of taxonomy and clinical symptoms. Clinically important pathogenic *Arcobacter* species include *Arcobacter butzleri, Arcobacter cryaerophilus*, and *Arcobacter skirrowii* [3]. Of these, *A. butzleri* is the most frequently isolated and associated with septicemia and gastroenteritis in humans [4]. In animals, the bacterium is primarily transmitted horizontally from the environment or one animal to another and vertically from parents to progeny [5]. Humans mainly get infected through ingestion and handling of fresh or undercooked contaminated foods of animal origin. Most *Arcobacter* infections are self-limiting and, hence, do not require treatment with antibiotics. Currently, tetracyclines and fluoroquinolones are the recommended antibiotics for treating infections caused by *Arcobacter* spp. [6].

In sub-Saharan Africa (SSA), the emergence of *Arcobacter* spp. resistant to tetracycline, aminoglycosides, and fluoroquinolones can be attributed to the excessive use of antibiotics in human medicine and animal husbandry [7–9]*.* Studies conducted in different geographical locations in SSA have reported multidrug-resistant *Arcobacter* [10, 11]. So far, more than 50 genes associated with tetracycline resistance in *Arcobacter* isolates from environmental samples have been described [12, 13]. Also, fluoroquinolone resistance associated with mutations in *gyrA* has been observed in *Arcobacter* species [14]. The World Health Organization (WHO) recently classified fluoroquinolone*-*resistant *Campylobacter*-like organisms as part of the 12 antibiotic-resistant priority pathogens that pose the greatest threat to human health [15].

Isolation of *Arcobacter* from local and imported poultry meat has been reported in Ghana [9]*.* In Ghana and Tanzania, poultry and livestock meat products are largely consumed, and most rural and semi-urban households own poultry [8]. Consumers may be at risk if farm animals carry pathogenic *Arcobacter* species*.* Monitoring and characterising *Arcobacter* species along the food chain is essential for a more accurate estimation of the population at risk. So far, only a few studies have been conducted in SSA, of which most studies focused on commercially produced poultry but not on the smallholder farm level [9, 16, 17]. Therefore, this study aimed to determine the frequency and antimicrobial resistance of *Arcobacter* species in both commercial and smallholder farm animals in Ghana and Tanzania.

## Results

### Frequency and species distribution of Arcobacter in smallholder and commercial farms

In Ghana, we sampled 15 commercial farms and 62 smallholder farms, while in Tanzania, we sampled 31 commercial farms and 71 smallholder farms. In total, 1585 samples were collected from farms in Ghana and 1047 from farms in Tanzania. The majority of samples from Tanzania were collected from smallholder farms (58.9%, n = 617), while in Ghana, the number of samples collected from commercial (50.8%, n = 805) and smallholder (49.2%, n = 780) farms were approximately the same. In both countries, chicken samples were the most frequently collected, making up 76.7% (n = 1216) of samples from Ghana and 74.2% (n = 777) from Tanzania. However, in Ghana, samples were also collected from other poultry birds such as turkey (n = 27), duck (n = 26), and quail (n = 28). Livestock samples in both countries were collected from cows (n = 271), goats (n = 138), pigs (n = 121), and sheep (n = 28). In total, 189 (11.9%, n/N = 189/1585) presumptive *Arcobacter* spp. were recovered from the samples collected from Ghana. In contrast, only 49 (4.7%) presumptive *Arcobacter* spp. were recovered from the samples collected from Tanzania. During freeze-storage, 5.8% (n = 11) of the presumptive *Arcobacter* spp. from Ghana and 38.8% (n/N = 19) from Tanzania were lost.

The relative frequency of confirmed *Arcobacter* spp. in poultry and livestock samples was higher in Ghana (84.1%, n/N = 111/132) than in Tanzania (15.9%, n/N = 21/132). The majority of the presumptive *Arcobacter* spp. that were not confirmed as *Arcobacter* spp. turned out to be *Campylobacter* spp. and *Comamonas* spp. Also, the relative frequency of the confirmed *Arcobacter* was higher in commercial farms in Ghana (87.4%, n/N = 83/95) compared to Tanzania (12.6%, n/N = 12/95). A total of eight different poultry (n = 4) and livestock (n = 4) species were sampled from commercial farms located in Ghana, and the incidence of *Arcobacter* was highest in pigs (45.1%, n/N = 37/82), followed by ducks (38.5%, n/N = 10/26), quails (35.7%, n/N = 10/28) and sheep (13.3%, n/N = 2/15). The remaining farm animal species had *Arcobacter* frequencies of less than 10%. The frequency of *Arcobacter* in chicken samples from commercial (3.7%, n/N = 20/545) and smallholder farms (4.0%, n/N = 27/671) in Ghana was similar. Table 1 provides details on the frequency of *Arcobacter* spp*.* isolated from poultry and livestock faecal samples collected from commercial and smallholder farms in Ghana and Tanzania.

**Table 1:** Frequency of *Arcobacter* spp*.* in commercial and smallholder farm animals in Ghana and Tanzania

Sample type	Commercial, % (n/N)		Smallholder, % (n/N)		Total, % (n/N)	
	Ghana	Tanzania	Ghana	Tanzania	Ghana	Tanzania
Chicken	3.7 (20/545)	3.2 (12/371)	4.0 (27/671)	1.2 (5/406)	3.9 (47/1216)	2.2 (17/777)
Turkey	7.4 (2/27)	NA	NA	NA	7.4 (2/27)	NA
Duck	38.5 (10/26)	NA	NA	NA	38.5 (10/26)	NA
Quail	35.7 (10/28)	NA	NA	NA	35.7 (10/28)	NA
Cow	1.5 (1/65)	0 (0/40)	NA	1.8 (3/166)	1.5 (1/65)	1.5 (3/206)
Pig	45.1 (37/82)	0 (0/19)	NA	0 (0/20)	45.1 (37/82)	0 (0/39)
Goat	5.9 (1/17)	NA	1.0 (1/98)	4.3 (1/23)	1.7 (2/115)	4.3 (1/23)
Sheep	13.3 (2/15)	NA	0 (0/11)	0 (0/2)	7.7 (2/26)	0 (0/2)
Total	10.3 (83/805)	2.8 (12/430)	3.6 (28/780)	1.5 (9/617)	7.0 (111/1585)	2.0 (21/1047)

*n* number positive, *N* total samples collected, and *NA* Not Applicable (No samples collected)

**Fig. 1:**
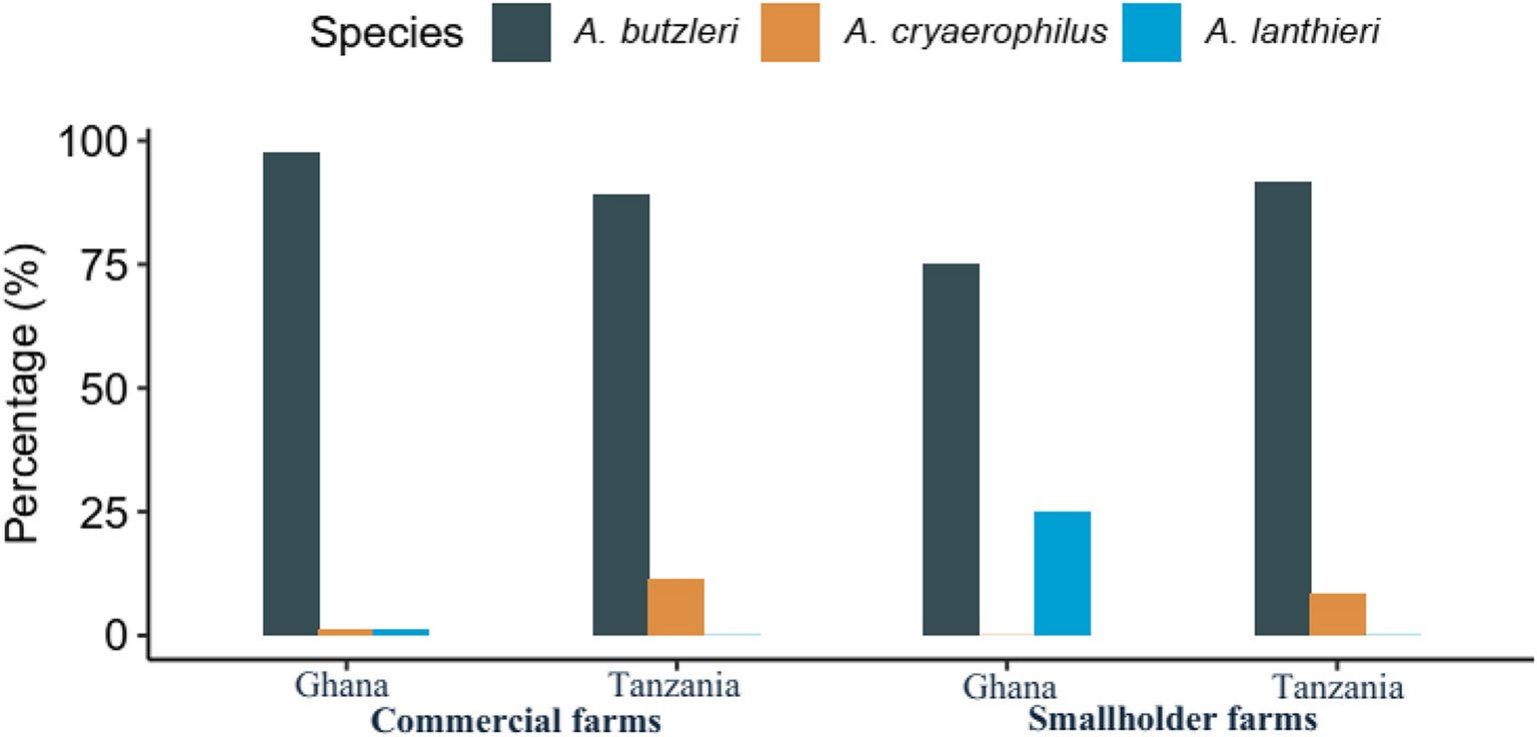
*Arcobacter* species from commercial and smallholder farms in Ghana and Tanzania

According to MALDI-TOF-based species identification, the majority of *Arcobacter* spp. isolated from both Ghana (91.9%, n/N = 102/111) and Tanzania (90.5%, n/N = 19/21) were identified as *A. butzleri*. The proportion of *A. butzleri* in commercial farms was similar to that of smallholder farms in Ghana and Tanzania. Three *A. cryaerophilus* were isolated, one from Ghana and two from Tanzania. All *Arcobacter lanthieri* (100%, n/N = 8/8) were isolated from chickens in Ghana, with the majority being isolated from smallholder farms (87.5%, n/N = 7/8) (Fig. 1).

### *Arcobacter* frequencies by month

**Fig. 2:**
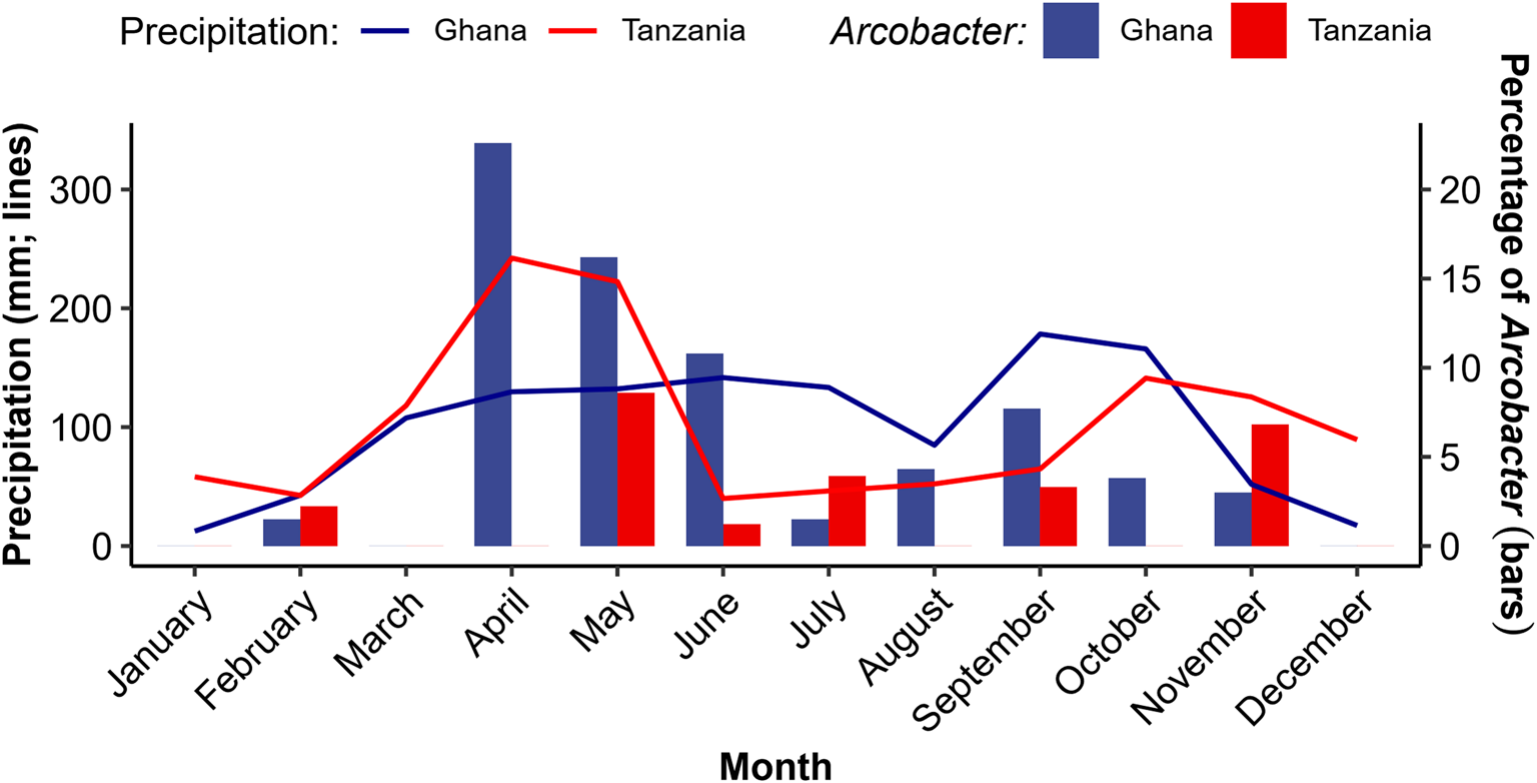
Monthly precipitation (line graph) and percentage of *Arcobacter* isolated (bar graph) from farms in Ghana and Tanzania. The monthly average precipitation data for the Tanga Region was acquired from (https://tcktcktck.org/tanzania/tanga/korogwe), whereas the data for the Ashanti Region was also obtained from (https://tcktcktck.org/ghana/ashanti)

The monthly precipitation (lines) and percentage of *Arcobacter* isolated (bars) from Ghana and Tanzania are shown in Fig. 2. Unlike Ghana, where *Arcobacter* was isolated in nine out of the 12 months of the year, in Tanzania, it was isolated in six out of the 12 months. *Arcobacter* was not isolated in both countries in January, March, and December. The monthly frequency in Ghana ranged from 0% to 22.6% in April. In May, Tanzania recorded the highest monthly frequency of 8.6% (n/N = 3/35). Almost all *Arcobacter* from Ghana (98.2%, n/N = 109/111) and 38.1% (n/N = 8/21) from Tanzania were isolated during the rainy season. In Ghana, *Arcobacter* were 20 times (95% CI 5.0–80.5) more likely to be isolated in the rainy season than during the dry season, while in Tanzania, detection rates were similar in both seasons (PR = 1.1, 95% CI 0.4–2.5).

### Antimicrobial resistance in *Arcobacter *species

Epidemiological cut-off values (ECOFFs) were derived for all antibiotics tested (Additional file 1). None of the *Arcobacter* isolates from smallholder farms in either country was resistant to tetracycline and kanamycin (Table 2). In contrast, 41.7% (n/N = 5/12) and 15.7% (n/N = 13/83) of *Arcobacter* isolates from commercial farms in Tanzania and Ghana, respectively, were resistant to tetracycline. Commercial farms from both countries were 5.2 (95% CI 1.7–15.8) and 4.7 (95% CI 1.2–18.8) times more likely to harbour ciprofloxacin and streptomycin*-*resistant *Arcobacter*, respectively, than smallholder farms. Of the eight antibiotics tested, ciprofloxacin exhibited the fourth-highest resistance level among isolates from Ghana (30.6%, n/N = 34/111) and Tanzania (42.9%, n/N = 9/21). *Arcobacter* from commercial farms in Tanzania was 5.9 (95% CI 2.4–14.7) and 2.7 (95% CI 1.2–6.1) times more likely to be resistant to erythromycin and tetracycline, respectively, than isolates from Ghana. Except for erythromycin, which showed a higher degree of resistance in Tanzania than Ghana isolates (PR = 2.9, 95% CI 1.3–6.3), all other antibiotics tested showed comparable resistance frequencies (Table 2).

**Table 2:** Antibiotic-resistant *Arcobacter* spp. isolated from commercial and smallholder farm animals in Ghana and Tanzania

Antibiotic	Resistance rate in % (n)					
	Commercial farm		Smallholder farm		Total	
	Ghana (N = 83)	Tanzania (N = 12)	Ghana (N = 28)	Tanzania (N = 9)	Ghana (N = 111)	Tanzania (N = 21)
Penicillin	100 (83)	100 (12)	100 (28)	100 (9)	100 (111)	100 (21)
Ampicillin	100 (83)	100 (12)	100 (28)	100 (9)	100 (111)	100 (21)
Chloramphenicol	100 (83)	100 (12)	100 (28)	100 (9)	100 (111)	100 (21)
Ciprofloxacin	39.8 (33)	58.3 (7)	3.6 (1)	22.2 (2)	30.6 (34)	42.9 (9)
Streptomycin	26.5 (22)	16.7 (2)	7.1 (2)	0 (0)	21.6 (24)	9.5 (2)
Erythromycin	8.4 (7)	50 (6)	21.4 (6)	11.1 (1)	11.7 (13)	33.3 (7)
Tetracycline	15.7 (13)	41.7 (5)	0 (0)	0 (0)	11.7 (13)	23.8 (5)
Kanamycin	12.0 (10)	8.3 (1)	0 (0)	0 (0)	9.0 (10)	4.7 (1)

*n* number positive and *N* total samples collected

All *A. lanthieri* isolates (100%, n/N = 8/8) were susceptible to ciprofloxacin, erythromycin, tetracycline, and kanamycin, and the majority (87.5%, n/N = 7/8) were susceptible to streptomycin. The observed resistance rates of *A. butzleri* (N = 121) to ciprofloxacin, streptomycin, erythromycin, tetracycline, and kanamycin were 33.9% (n = 41), 19.0% (n = 23), 15.7% (n = 19), 13.2% (n = 16), and 8.3% (n = 10), respectively.

**Fig. 3:**
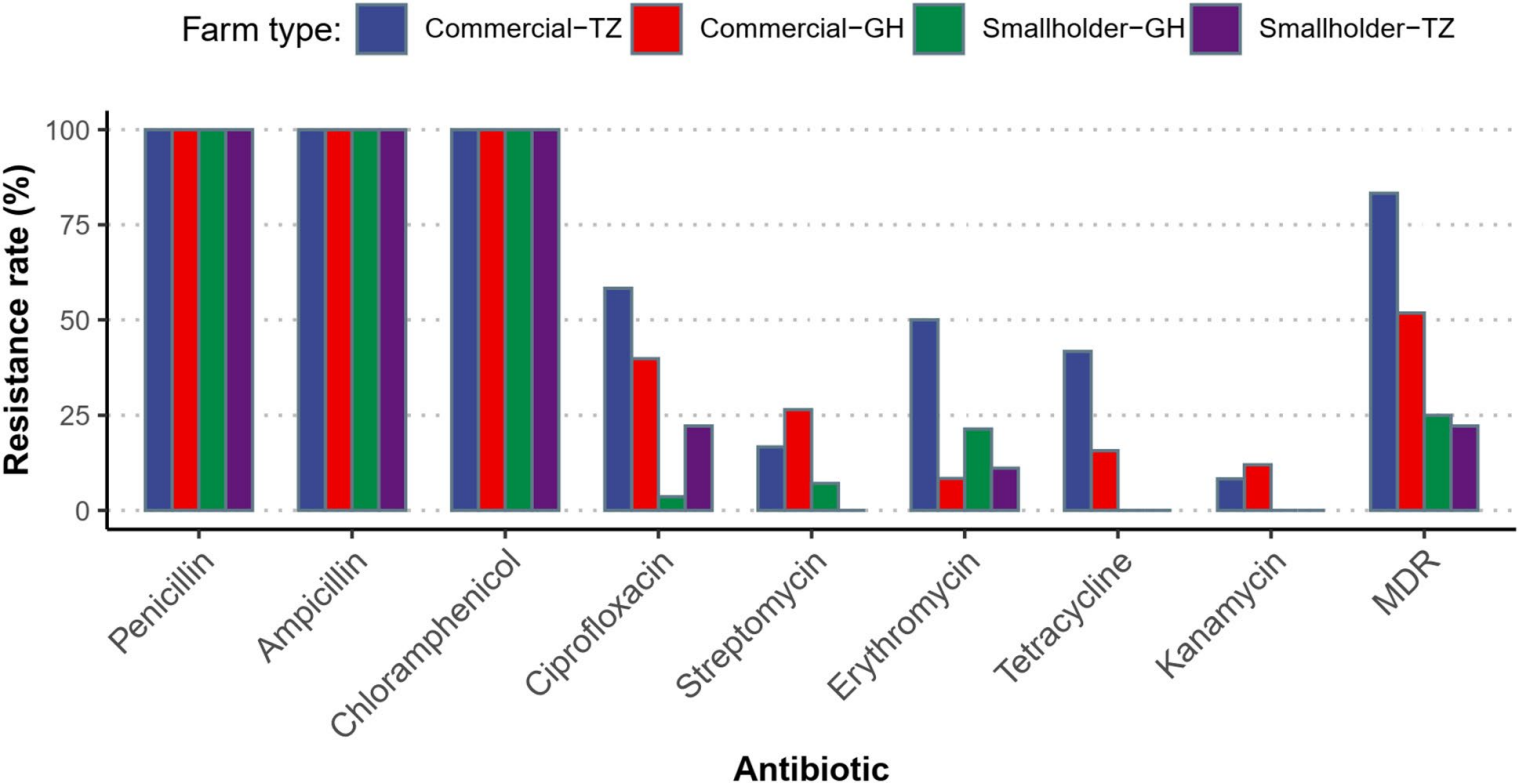
Antibiotic resistance of *Arcobacter* isolates from commercial and smallholder farms in Tanzania and Ghana. *TZ* Tanzania, *GH* Ghana, *MDR* multi drug resistance

Figure 3 shows antibiotic resistance of *Arcobacter* isolates from commercial and smallholder farms in Tanzania and Ghana. In general, higher antibiotic resistance was observed in *Arcobacter* from commercial farms compared to smallholder farms in both countries. Also, more resistant isolates were observed in *Arcobacter* from commercial farms in Tanzania than in Ghana. Multi-drug resistance (MDR) was observed in 57.1% (n/N = 12/21) and 45.0% (n/N = 50/111) of *Arcobacter* isolates from Tanzania and Ghana, respectively.

Multidrug resistance (MDR) was observed in 57.1% (n/N = 12/21) and 45.0% (n/N = 50/111) of the *Arcobacter* isolates from Tanzania and Ghana, respectively. Table 3 summarizes the factors associated with MDR in all *Arcobacter* isolates. The type of farm (commercial or smallholder) and source of the sample (poultry or livestock) were found to be associated with MDR (Table 3). In both countries combined, a higher prevalence of MDR *Arcobacter* was isolated from commercial farms (55.8%, n/N = 53/95) than from smallholder farms (24.3%, n/N = 9/37) (PR = 2.3, 95% CI 1.3–4.2). The adjusted PRs also indicate that poultry were 1.3 times (95% CI 1.1–2.6) more likely to have MDR *Arcobacter* strains than livestock. However, seasonal variation, the country from which samples were collected, and the particular *Arcobacter* species were not associated with MDR.

**Table 3:** Associations with the frequency of multi drug-resistant *Arcobacter*

Variable	Crude PR (95% CI)	Adjusted PR (95% CI)
Commercial vs. smallholder farm	2.3 (1.3–4.2)	2.7 (1.6–4.9)
Rainy vs. dry season	1.3 (0.3–2.0)	1.3 (0.3–1.8)
Poultry vs. livestock	1.6 (0.9–1.5)	1.3 (1.1–2.6)
*Arcobacter butzleri* vs. Other species	1.8 (0.7–4.7)	2.1 (0.8–5.5)
Ghana vs. Tanzania	0.8 (0.5–1.2)	0.9 (0.6–1.3)

*PR* prevalence ratio, *CI* confidence interval

## Discussion

The present study describes antibiotic-resistant *Arcobacter* species from commercial and smallholder farm animals in Ghana and Tanzania. The observed overall *Arcobacter* proportion in Ghana (7.0%) and Tanzania (2.0%) was much lower than what was described in a previous study with a focus on local and imported poultry meat in Kumasi, Ghana (26.5%) [9] and a study conducted in ostriches in South Africa (68%) [18], and in poultry abattoir effluents in Nigeria (14.0%) [17]. The differences in the current *Arcobacter* proportion compared to the few earlier studies conducted in similar geographical areas could be due to several factors. For instance, the types of samples analyzed, variations in the timing of sample collection throughout the year and the specific microbiological methods utilized. Among the different farm animals sampled in Ghana, pigs (45.1%), ducks (38.5%), and quails (35.7%) had the highest overall *Arcobacter* frequencies. While a few studies have reported similar findings [19, 20], other studies conducted in Asia and Africa have observed the highest *Arcobacter* frequencies in chicken [3, 11, 18]

In this study, Matrix-Assisted Laser Desorption Ionization Time-of-Flight Mass Spectrometry (MALDI-TOF MS) species identification revealed the presence of three types of *Arcobacter* spp.: *A. butzleri*, *A. cryaerophilus*, and *A. lanthieri*. The predominant species was *A. butzleri*, which is not uncommon in poultry and livestock [20–22] and is also most commonly implicated in human infections. Surprisingly, *A. skirrowii* was not found in this study, even though it is a known colonizer of poultry and livestock [11, 23]. The present study identified eight *A. lanthieri* from chicken in Ghana with the majority being isolated from smallholder farms. *A. lanthieri* was only recently described in 2015 [24] and since then, it has been isolated from pigs, dairy cattle manure, and humans [24–26]. The presence of *A. lanthieri* in farms in Ghana is concerning as it is known to encode many putative virulence genes [25].

In this study, the isolation rate for *Arcobacter* in Ghana was much higher in the rainy season than in the dry season, while in Tanzania, the detection rate was similar in both seasons. In temperate climates, there is no consensus on the differences in *Arcobacter* prevalence by season. A recent study observed varied frequencies according to season and poultry type [20]. Similarly, studies conducted in Japan and Italy recorded no significant difference in prevalence by season [3, 27]. However, in tropical settings, higher frequencies of enteric bacterial pathogens have been observed in the rainy season than in the dry season [28, 29]. The significantly higher contamination of farms in Africa by enteric bacterial pathogens during the rainy season has been attributed to open defecation practices, increased runoff, and more frequent overflowing of onsite septic tanks and sanitation systems [30]. In addition, the lower temperatures during the rainy seasons favour the survival of mesophilic foodborne pathogens such as *Arcobacter.*

Because no ECOFF values could be defined for penicillin, ampicillin and chloramphenicol due to the lack of discrimination of distinct susceptible or resistant isolate populations, all *Arcobacter* isolates tested in this study were considered resistant to these three antibiotics. This is in line with studies conducted in Turkey and Iran, where most *Arcobacter* isolates were found to be resistant to ampicillin and chloramphenicol, respectively. [10, 11]. Also, 32.6% and 19.7% of the *Arcobacter* isolates tested against ciprofloxacin and streptomycin, respectively, had inhibition zone diameters below the ECOFF values indicating resistance for both antimicrobials. A recent study on backyard chickens and retail poultry meat in Chile found lower rates of ciprofloxacin, tetracycline, and erythromycin resistance [31]. The increased resistance rate observed in this study could be due to differences in geographic location and misuse of antibiotics in commercial and smallholder farms in the current study areas [8, 32]. Not surprisingly, the resistance patterns of *Campylobacter* isolates from farms in the study area in Ghana showed similar results to those reported here [28, 33]. Nevertheless, it is reassuring that our study observed that all *Arcobacter* spp. from smallholder farms in the two countries were susceptible to both tetracycline and kanamycin. This could be due to the lower use of antibiotics in smallholder farms compared to commercial farms, as previously described in the same study area in Ghana [8].

*A. butzleri* was found to be generally more resistant to antibiotics than *A. lanthieri.* This correlates with findings from previous studies [34, 35]. Among all known *Arcobacter* spp., *A. butzleri* has been reported as the most significant clinical pathogen due to its high overall prevalence and pathogenicity [35]. We also identified multidrug-resistant *Arcobacter* spp. in this study. The inherent resistance of Campylobacteraceae to β-lactam antibiotics may explain the high resistance rate observed [2]. We observed more multidrug-resistant *Arcobacter* isolates in poultry than in livestock. A report from Tanzania suggests that antimicrobial misuse is widespread among farmers, with poultry farmers having higher rates of misuse than livestock farmers [36].

There were some limitations in our study. Sampling was limited to a single district in both countries, so the observed results may not reflect true nationwide prevalence in each country. The high number of presumptive isolates from Tanzania dying during freeze storage resulting in low *Arcobacter* frequencies, and the less variety of farm animals sampled from Tanzania, made it difficult to do a detailed comparison between the two countries but rather show trends only. In addition, the enrichment and selective medium used in this study disproportionately favour the isolation of *A. butzleri*, probably at the expense of other *Arcobacter* species*.* Despite the above limitations, this study is, to the best of our knowledge, the first to report on *Arcobacter* species in both commercial and smallholder farms in Ghana and Tanzania.

## Conclusion

Our findings suggest that commercial and smallholder farm animals in Ghana and Tanzania are carriers and potential transmission reservoirs for *Arcobacter* species. All *Arcobacter* recovered from poultry and livestock were resistant to at least two or more antibiotic classes tested. The high levels of MDR *Arcobacter* detected call for immediate development and implementation of effective *Arcobacter* control strategies in commercial and smallholder farms to curb the proliferation of multidrug-resistant strains and safeguard animal and human health. Furthermore, our findings may inspire further research in SSA to comprehensively understand the prevalence, virulence, and pathogenicity of *Arcobacter* spp. across a broader range of geographic areas.

## Methods

### Study site

**Fig. 4:**
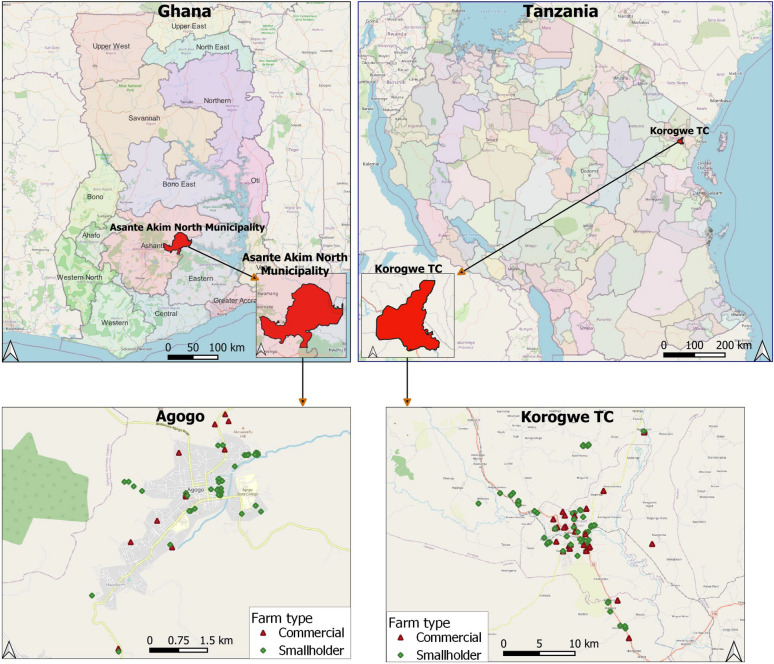
Location of commercial and smallholder farms in Agogo, Ashanti Region, Ghana and Korogwe TC, Tanga Region, Tanzania that were included in the study. This map was created using the QGIS version 3.24.0-Tisler software (https://qgis.org/en/site/)

A cross-sectional study was conducted in two countries in SSA, Ghana and Tanzania. In Ghana, this study was conducted in Agogo, the capital of the Asante Akim North Municipality, located in the eastern part of the Ashanti Region (Fig. 4). Asante Akim North Municipality is a rural community with a population of 85,788 [37]. Almost half (42%) of the households in the municipality rear farm animals, and poultry accounts for 56% of the animals, with the remaining ones being livestock [37]. Ghana has a tropical climate with two main seasons. The rainy season extends from April to October, and the dry season from November to March.

In Tanzania, this study was conducted in Korogwe Town Council (TC), located within the Tanga Region in northeastern Tanzania (Fig. 4). Based on preliminary results of the 2022 Tanzania population and housing census [38], Korogwe TC population is estimated at 73,464. Tanzania has a tropical Savannah climate with two rainy seasons. March to May is characterized by long rains, and November to mid-January by short and lighter rains. Most of the population resides in rural settings, mainly engaging in informal trade or subsistence farming (hereafter called smallholder farming).

### Sample collection

Sampling took place between March 2019 and July 2020. A farm with an intensive housing system of caged poultry and/or livestock was considered commercial. Smallholder farms were households with free-roaming poultry (mainly indigenous breeds) and/or livestock with shelter provided by basic or temporary roofing. Before sampling, a list of all commercial farms within each study site was obtained from each country’s respective district office of the Ministry of Agriculture. In a community within the study site, we initially identified one or two households engaged in rearing free-range farm animals. We then requested those households to introduce us to another household that kept farm animals for possible sampling. Before sample collection, the farm was visited to ascertain the number of pen houses. Multiple pen house farms were visited more than once during sampling; nonetheless, each pen house was sampled only once throughout the study period. Faecal samples were collected from poultry and livestock in the commercial and smallholder farms. Poultry included chicken, duck, turkey, and quail, while livestock included sheep, goats, pigs, and cows. For each sample, approximately 2 *g* of freshly voided faecal droppings were collected using a sterile spatula and placed in a sterile plastic container without preservatives. All samples were transported in a cool box (4–8 ℃) and processed within 2–4 h at the Kumasi Centre for Collaborative Research in Tropical Medicine (KCCR) in Ghana or the National Institute for Medical Research (NIMR) in Korogwe, Tanzania.

### Identification of Arcobacter

*Arcobacter* spp. was isolated using selective enrichment media as described by [10]. Suspected *Arcobacter* colonies were tested for the enzyme cytochrome oxidase and those that were positive were examined by Gram staining. Gram-negative spiral-rod-shaped colonies were stored, as presumptive *Arcobacter* isolates, at −80 ℃ using the Microbank^™^ system (Pro-Lab Diagnostics, Bromborough, UK). All isolates were shipped to Germany on dry ice and species confirmation was performed by MALDI-TOF MS using the VITEK^®^ MS system (bioMérieux, Marcy-l'Étoile, France).

### Antibiotic susceptibility testing

The Kirby Bauer disk diffusion method [39] was used to assess the antibiotic susceptibility of all confirmed *Arcobacter* isolates. Antibiotic disks (Oxoid, Hampshire, UK) were placed on Mueller–Hinton agar supplemented with 5% sheep blood and inoculated with *Arcobacter* for antibiotic susceptibility testing. Plates were incubated at 30 ℃ under microaerophilic conditions for 24 h. After 24 h, isolates with insufficient growth were further incubated, and the inhibition zone was read after a total of 40–48 h. Isolates were tested against ampicillin (10 µg), chloramphenicol (30 µg), ciprofloxacin (5 µg), streptomycin (25 µg), erythromycin (15 µg), tetracycline (30 µg) and kanamycin (30 µg). So far, the European Committee on Antimicrobial Susceptibility Testing (EUCAST) clinical breakpoints have not been determined for *Arcobacter*, therefore, ECOFFs were determined based on the frequency distribution of measured inhibition zone diameters (Additional file 1). Additional *Arcobacter* isolates obtained from children at the same study sites during the research period were included in the development of the ECOFFs. (Additional file 1). The procedure for developing ECOFFs has been described previously [40, 41]. The zone diameter measurements, indicating susceptibility (S) or resistance (R) for each antibiotic, are detailed in Table 4. Multidrug resistance (MDR) was defined as resistance to at least one agent in three or more antimicrobial categories.

**Table 4:** Epidemiological Cut-Off Values (ECOFFs) used for Antimicrobial Resistance in *Arcobacter* spp

Antibiotic (disk concentration)	Zone diameter (mm)	
	S ≥	R <
Tetracycline (30 µg)	18	18
Ciprofloxacin (5 µg)	18	18
Streptomycin (25 µg)	15	15
Ampicillin (10 µg)	NA	NA
Chloramphenicol (30 µg)	NA	NA
Erythromycin (15 µg)	11	11
Kanamycin (30 µg)	14	14
Penicillin (10 µg)	NA	NA

*S* susceptible, *R* resistant, *NA* not applicable—100% resistant

### Data analysis

Descriptive statistics of categorical variables were calculated using absolute frequencies and corresponding percentages. Prevalence ratios (PRs) and their respective 95% confidence intervals (CIs) were computed to show associations between two categorical variables. Because of the explanatory nature of this study, p-values were not calculated. Poisson regression with robust standard errors was used to fit multivariable models for multiple drug resistance in *Arcobacter* isolates. The dependent variable in the Poisson regression was whether an *Arcobacter* isolate was multiple drug-resistant or not. The independent variables were whether the isolate was collected from a commercial or smallholder farm, during the rainy or dry season, from poultry or livestock samples, and coming from Ghana or Tanzania. R software (version 4.3.1) was used for all statistical analyses [42]. The *epiR* (2.0.19) package was used to calculate the PRs, and the *sandwich* package (version 3.0–0) was used to compute robust standard errors of the Poisson regression. A bar chart was created, using the R package *ggplot*2 (version 3.3.5), to show *Arcobacter* spp. with inhibition zone diameters below (resistant) and above (susceptible) the ECOFFs. Also, the line graph and bar chart showing *Arcobacter* frequency by month were plotted using the *ggplot2* package (version 3.3.5). The line graph for the Tanga Region was plotted using the monthly average precipitation data obtained from https://tcktcktck.org/tanzania/tanga/korogwe, whereas the data for the Ashanti Region was also acquired from https://tcktcktck.org/ghana/ashanti/agogo. QGIS software, version 3.24 [43], was used to draw a map showing the geographical location of the farms sampled in Ghana and Tanzania.

### Supplementary Information

**Additional file 1.** Epidemiological cut-off values (ECOFFs) determined based on the frequency distribution of measured inhibition zone diameters of all antibiotics tested against *Arcobacter* isolates

## Data Availability

The raw data supporting the conclusions of this article are included in the article or are available as supplementary data files.
